# Acute Kidney Injury Biomarkers in Marathon Runners: Systematic Review and Meta-Analysis

**DOI:** 10.3390/medicina61101775

**Published:** 2025-10-01

**Authors:** Daniel-Corneliu Leucuța, Loredana-Ioana Trif, Oana Almășan, Ștefan Lucian Popa, Abdulrahman Ismaiel

**Affiliations:** 1 Department of Medical Informatics and Biostatistics, Iuliu Hațieganu University of Medicine and Pharmacy, 400349 Cluj-Napoca, Romania; dleucuta@umfcluj.ro (D.-C.L.);; 2 Department of Prosthetic Dentistry and Dental Materials, Iuliu Hațieganu University of Medicine and Pharmacy, 32 Clinicilor Street, 400006 Cluj-Napoca, Romania; 3 2nd Department of Internal Medicine, Iuliu Hațieganu University of Medicine and Pharmacy, 400006 Cluj-Napoca, Romania

**Keywords:** marathon, acute kidney injury, biomarkers

## Abstract

*Background and Objectives*: The objectives of this review were as follows: to measure changes in renal biomarker levels before, immediately after, and 24 h post-marathon; to identify promising biomarkers for the diagnosis of acute kidney injury; and to describe the temporal patterns of biomarker dynamics in relation to the marathon. *Materials and Methods*: Studies of marathon runners reporting AKI-related biomarkers were included. Four databases (PubMed, EMBASE, Web of Science, and LILACS) were searched. Data on study design, participant characteristics, and biomarker values (pre-, post-, and 24 h post-race) were extracted, and a random effects meta-analysis was performed. Risk of bias was assessed with the National Heart, Lung, and Blood Institute pre–post tool. *Results*: The study showed significant increases in most biomarkers immediately after the marathon compared to baseline values. The largest increases were observed in Tissue Inhibitor of Metalloproteinases-2* Insulin-like Growth Factor Binding Protein-7 (TIMP-2*IGFBP), copeptin, urinary Liver-type Fatty Acid Binding Protein (L-FABP), urinary Monocyte Chemoattractant Protein-1 (MCP-1), IGFBP-7, urinary Chitinase 3-like Protein 1 (YKL-40), and TIMP-2, suggesting that these biomarkers are promising candidates for future research. Several patterns of biomarker evolution were observed: some increased without decreasing even at 24 h after the marathon; others increased post-marathon and decreased at 24 h while remaining above baseline; some increased after the marathon and then fell below baseline at 24 h. *Conclusions*: Marathon running causes significant increases in kidney injury biomarkers, with different patterns of evolution.

## 1. Introduction

Recently, running has gained popularity [1,2,3]. Long-distance running is one of the forms of running, and can include half marathons, marathons, and ultramarathons. Physical activity is acknowledged as a health-protective factor [2,4]. Nevertheless, long-distance running can induce high stress on different organs and systems [5,6]. Studies have found out that the kidneys can be affected during a marathon. Some runners may experience stage 1 acute kidney injury (AKI) [7,8]. AKI is defined as an unexpected and often temporary decline in kidney function, as indicated by a rise in creatinine or a fall in urine volume [9]. Stage 1 AKI is defined by KDIGO as a rise in serum creatinine by ≥0.3 mg/dL within 48 h, or a rise up to 1.5–1.9 times baseline within the prior 7 days, or a decrease in urine excretion <0.5 mL/kg/h for 6–12 h [10]. These episodes are mainly reversible, but when repeated, or maybe in cases with comorbidities or in the presence of other risk factors, a long-term renal injury can happen [11]. It is yet uncertain if recurrent renal insults that fit the criteria of AKI cause an accelerated development of long-term renal problems in long-distance running [12]. In addition to serum creatinine and urine output, there are many biomarkers for AKI. A classification by Oh et al. [13] identifies three main categories: functional biomarkers: serum creatinine, serum cystatin C (sCys C); tubular enzymes; damage biomarkers: urinary Neutrophil Gelatinase-Associated Lipocalin (uNGAL), and serum NGAL (sNGAL), urinary Kidney Injury Molecule-1 (uKIM-1), Interleukin 18, urinary liver-type fatty acid binding protein (uL-FABP); and pre-injury phase biomarkers: urinary Tissue Inhibitor of Metalloproteinases-2 (uTIMP-2) and urinary Insulin-like Growth Factor Binding Protein-7 (uIGFBP-7).

Although many observational studies have shown biomarker modifications after a marathon, no systematic synthesis has assessed the biomarkers’ temporal patterns and diagnosis over time.

The study aimed to investigate changes in urinary, serum, and plasma biomarkers indicative of acute kidney injury in individuals participating in marathons. The objectives were as follows: to measure changes in renal biomarker levels before, immediately after, and 24 h post-marathon; to identify promising biomarkers for the diagnosis of acute kidney injury; and to describe the temporal patterns of biomarker dynamics in relation to the marathon.

## 2. Materials and Methods

The present manuscript followed the “Preferred Reporting Items for Systematic Reviews and Meta-analyses Protocols (PRISMA)” [14].

### 2.1. Eligibility Criteria

We included prospective and retrospective observational studies involving (P) individuals who participated in (I) running a marathon, with the primary outcome (O) being the changes in urinary, serum, or plasma biomarkers that may be indicative of acute kidney injury (serum creatinine, urinary creatinine, BUN-to-creatinine ratio, serum urea, TIMP-2, IGFBP-7, TIMP-2*IGFBP, urinary L-FABP, urinary NGAL, plasma NGAL, serum cystatin C, plasma KIM-1, urinary KIM-1, plasma TNF-alpha, urinary TNF-alpha, plasma MCP-1, urinary MCP-1, plasma YKL-40, and urinary YKL-40). Secondary outcomes included serum C-reactive protein, copeptin, and serum creatine kinase. Studies involving other types of marathons (e.g., half-marathon, ultramarathon, or other distances), as well as reviews, meta-analyses, editorials, letters to the editor, and conference abstracts, were excluded.

### 2.2. Information Sources

To identify studies that met our selection criteria, we searched four databases: PubMed, EMBASE, Web of Science, and LILACS. Additionally, the reference lists of the selected articles and reviews were examined to identify further relevant studies.

### 2.3. Search Strategy

The search strategy included the following terms: acute kidney injury and marathon, as well as the relevant biomarkers, using MeSH terms, synonyms, singular and plural forms, and abbreviations. The search was conducted from inception until 29 May 2024. No language restrictions were applied in the search strategies or in the selection of articles. The complete search strategy for each database is provided in Supplementary Table S1.

### 2.4. Selection Process

An initial semi-automated removal of duplicate studies was performed using Zotero version 7.0.11 (Corporation for Digital Scholarship, Vienna, VA, USA) [15]. Subsequently, titles and abstracts were manually screened to exclude articles that did not meet the eligibility criteria, as well as any remaining duplicates. Full-text versions of the remaining articles were then manually reviewed by two authors (D.-C.L., L.-I.T.), and irrelevant studies, non-eligible article types, and duplicates were excluded.

### 2.5. Data Collection Process

From each selected study, data was manually extracted by several authors (D.-C.L., L.-I.T., O.A., Ș.L.P., and A.I.). The information included study characteristics, country, region, marathon location, study design, age, percentage of female marathon participants (sex), body mass index, the marathon event, previous marathons completed, weekly running distance (km/week), running history (years), AKI criteria, AKI stage 1 reporting, and values of urinary, serum, and plasma biomarkers, and indicators of acute kidney injury.

### 2.6. Effect Size

For each outcome, the mean and standard deviation were extracted. When this data was unavailable, values were estimated from the median and interquartile range using formulas provided in the Cochrane Handbook [16]. The effect size of interest was represented by the mean values of the biomarkers. Measurements were collected at three time points: before the marathon, immediately after the marathon, and 24 h post-marathon.

### 2.7. Risk of Bias Assessment

Several authors assessed the methodological quality (D.-C.L., O.A., Ș.L.P., and A.I.) of the selected articles with the quality assessment tool for before and after (pre–post) studies with no control group from the National Heart, Lung, and Blood Institute (NHLBI) [17]. In addition to the questions in the NHLBI assessment tool, we added 4 supplementary original questions to broaden and clarify the risk of bias assessment: Clear exclusion criteria for the following: alcohol or food abstinence 12 h before baseline measurement; exclusion of comorbidities that could alter measurements; exclusion of medication that could alter measurements; and presenting the use of water, electrolytes, or food during the marathon.

### 2.8. Synthesis Methods

Means and standard deviations were entered into meta-analyses using the meta package in R [18]. Due to clinical heterogeneity among studies, the mean and 95% confidence intervals (CIs) for each variable were calculated using a random-effects model. Results were presented as forest plots. The chi-squared-based Q test and the I^2^ statistic were used to assess statistical heterogeneity between studies. Statistical significance was defined as a *p*-value less than 0.05. All analyses were performed using R statistical software, version 4.3.2 (R Foundation for Statistical Computing, Vienna, Austria) [19].

### 2.9. Assessment of Publication Bias

Publication bias was assessed using Egger’s test.

During the preparation of this manuscript, the authors used ChatGPT version 4.0 (OpenAI, San Francisco, CA, USA, accessed in June 2025) as a brainstorming tool to generate suggestions for perspectives to include in the Discussion section. Also, it was used to improve the scientific writing of the manuscript. The authors have reviewed and edited the output and take full responsibility for the content of this publication.

A protocol for the publication can be found at protocols.io with the ID 225510 (registered on 26 August 2025).

## 3. Results

**Figure 1:**
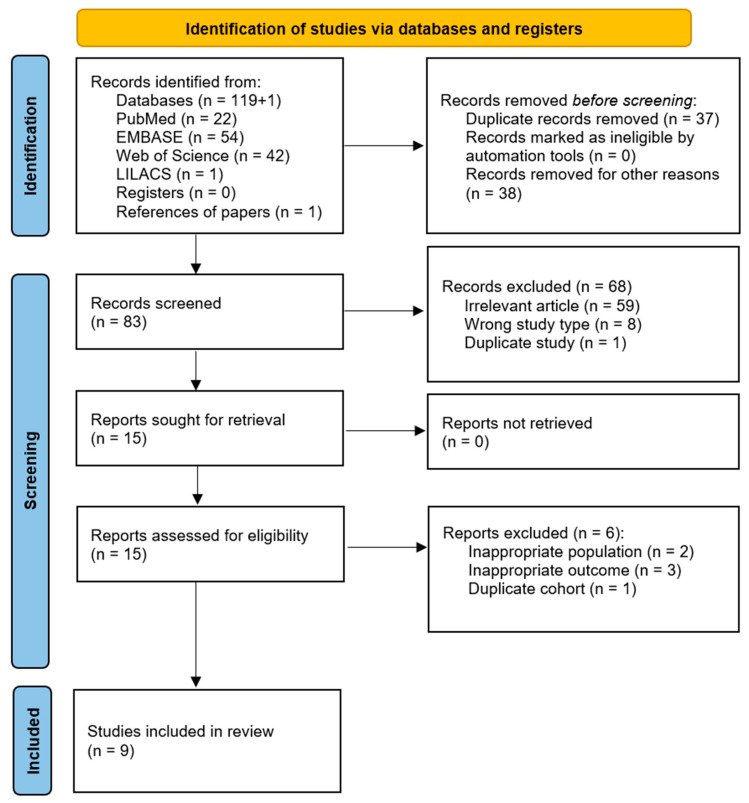
Flow chart illustrating the identification, selection, and inclusion of articles in the review.

A total of 119 records were retrieved from the four databases searched, and one additional study was identified through reference screening of the selected articles. The identification and selection process is illustrated in Figure 1. After removing 37 duplicates, 83 records were screened based on title and abstract. Of these, 68 were excluded for not meeting the eligibility criteria or being duplicates. The full texts of the remaining 15 articles were then assessed, resulting in the inclusion of 9 studies in the final review and meta-analysis.

### 3.1. Study Characteristics

The characteristics of the included studies are detailed in Table 1 and Table 2. Five studies were conducted in Europe, and four in America. All studies employed a prospective cohort design. Acute kidney injury among marathon runners was diagnosed based on the RIFLE, AKIN, or KDIGO criteria. The mean age of participants varied across studies, with several reporting a mean age of around 40 years. However, one study included younger participants with a mean age of 23 years, while another reported a higher mean age of 50 years. Two studies included only male participants. In five studies, the proportions of male and female participants were approximately equal, while in two others, the percentage of female participants was 18% and 29%, respectively. Previous marathon experience, weekly running distance, and running history (in years) also varied across the studies.

### 3.2. Most Frequently Reported Biomarkers

**Figure 2:**
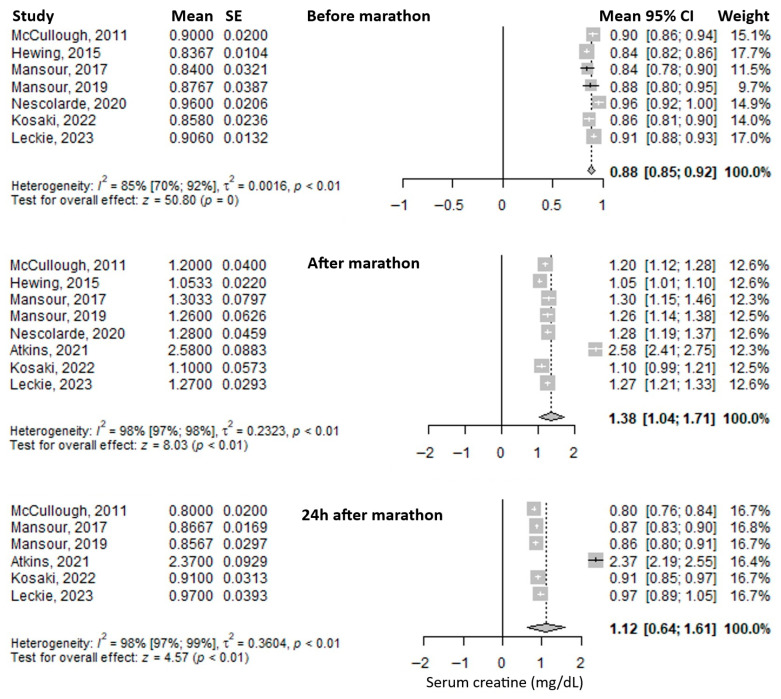
Forest plots of the mean serum creatinine values before the marathon, immediately after the marathon, and 24 h after the marathon. Before marathon [7,8,20,21,23,24,26], after marathon [7,8,20,21,22,23,24,26], 24 h after marathon [7,8,20,21,22,24].

Serum creatinine was the most frequently reported biomarker. Its mean value before the marathon was 0.88 mg/dL (95% CI: 0.85–0.92), it increased immediately after the marathon to 1.38 mg/dL (95% CI: 1.04–1.71), and decreased at 24 h post-marathon to 1.12 mg/dL (95% CI: 0.64–1.61) (Figure 2). Heterogeneity of the results was substantial, with I^2^ inconsistency indices of 85%, 98%, and 98% for the three respective time points, all statistically significant.

**Figure 3:**
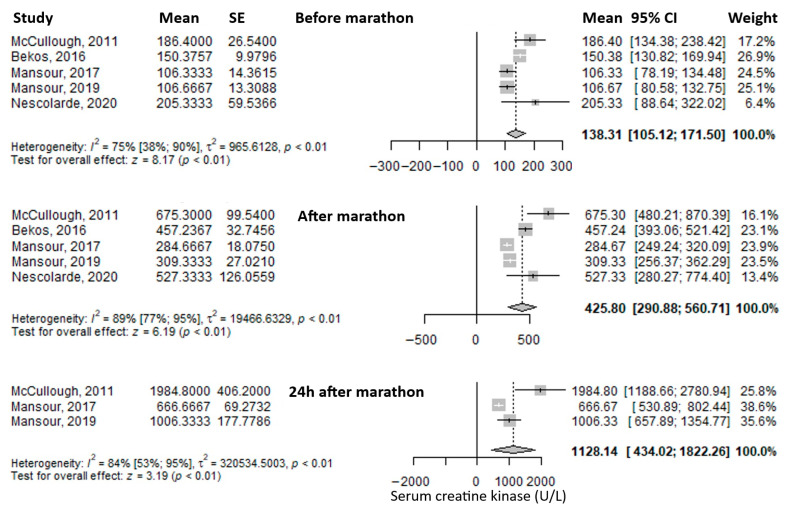
Forest plots of the mean serum creatin kinase values before the marathon, immediately after the marathon, and 24 h after the marathon. Before marathon [7,8,23,24,25], after marathon [7,8,23,24,25], 24 h after marathon [7,8,24].

Another frequently reported biomarker was serum creatine kinase. The mean value before the marathon was 138.31 U/L (95% CI: 105.12–171.5), which increased threefold immediately after the marathon to 425.8 U/L (95% CI: 290.88–560.71), and rose even further at 24 h post-marathon—approximately an eightfold increase compared to baseline—to 1128.14 U/L (95% CI: 434.02–1822.26) (Figure 3). Heterogeneity was substantial, with I^2^ inconsistency values of 75%, 89%, and 84% for the three respective time points, all statistically significant.

**Figure 4:**
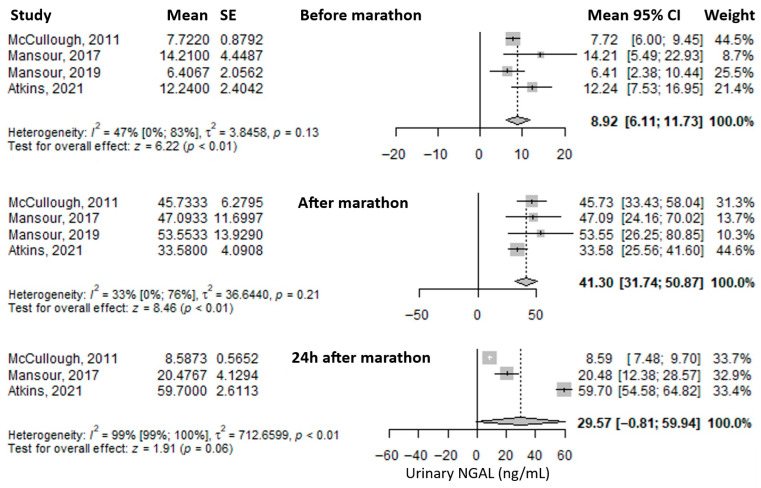
Forest plots of the mean urinary Neutrophil Gelatinase-Associated Lipocalin (NGAL) values before the marathon, immediately after the marathon, and 24 h after the marathon. Before marathon [7,8,22,24,25], after marathon [7,8,22,24,25], 24 h after marathon [7,22,24].

The most frequently reported urinary biomarker was urinary Neutrophil Gelatinase-Associated Lipocalin (uNGAL). The mean value before the marathon was 8.92 ng/mL (95% CI: 6.11–11.73), which increased substantially—approximately fourfold—immediately after the marathon to 41.30 ng/mL (95% CI: 31.74–50.87), and decreased to 29.57 ng/mL (95% CI: –0.81 to 59.94) at 24 h post-marathon, remaining approximately three times higher than baseline (Figure 4). Heterogeneity was moderate before and immediately after the marathon (I^2^ = 47% and 33%, respectively), and considerable at 24 h post-marathon (I^2^ = 99%), with only the latter being statistically significant.

**Figure 5:**
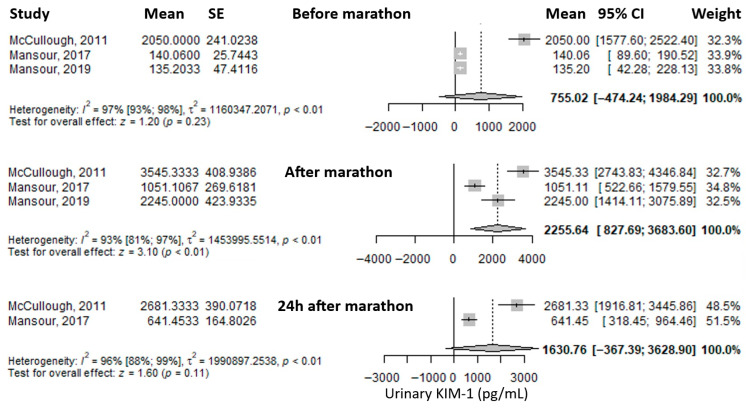
Forest plots of the mean urinary Kidney Injury Molecule-1 (KIM-1) values before the marathon, immediately after the marathon, and 24 h post-marathon. Before marathon [7,8,24], after marathon [7,8,24], 24 h after marathon [7,24].

Another reported urinary biomarker was urinary Kidney Injury Molecule-1 (uKIM-1). The mean value before the marathon was 755.02 pg/mL (95% CI: –474.24 to 1984.29), which increased significantly—approximately threefold—immediately after the marathon to 2255.64 pg/mL (95% CI: 827.69 to 3683.6), and then decreased at 24 h post-marathon to 1630.76 pg/mL (95% CI: –367.39 to 3628.9), remaining about twice as high as baseline (Figure 5). Heterogeneity of the results was considerable at all three time points, with I^2^ values of 97%, 93%, and 96%, respectively, all statistically significant.

**Figure 6:**
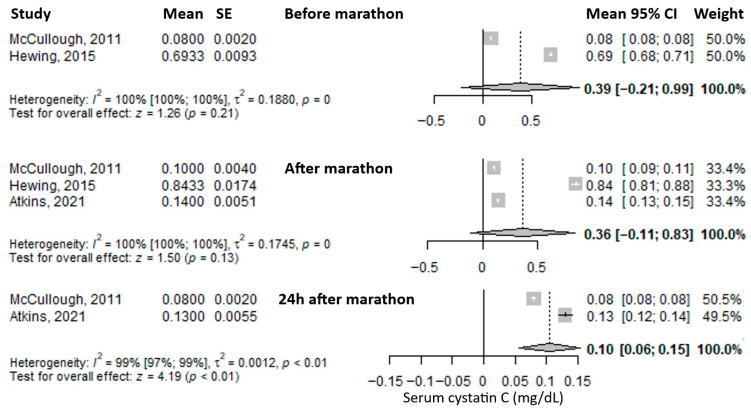
Forest plots of the mean serum cystatin C values before the marathon, immediately after the marathon, and 24 h post-marathon. Before marathon [24,26], after marathon [22,24,26], 24 h after marathon [22,24].

Another reported biomarker was serum cystatin C. The mean value before the marathon was 0.39 mg/L (95% CI: –0.21 to 0.99), followed by a slight decrease immediately after the marathon to 0.36 mg/L (95% CI: –0.11 to 0.83), and a further, more pronounced decrease at 24 h post-marathon to 0.10 mg/L (95% CI: 0.06–0.15), representing roughly one-quarter of the baseline level (Figure 6). Heterogeneity was considerable at all three time points, with I^2^ values of 100%, 100%, and 99%, respectively—all statistically significant.

**Figure 7:**
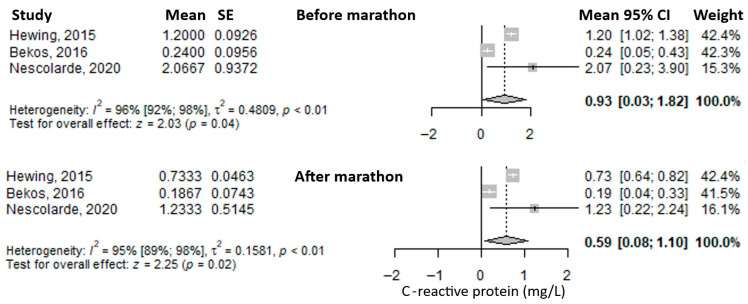
Forest plots of the mean serum C-reactive protein values before the marathon and immediately after the marathon. Before marathon [23,25,26], after marathon [23,25,26].

Another reported biomarker was C-reactive protein (CRP). The mean value before the marathon was 0.93 mg/L (95% CI: 0.03–1.82), which decreased immediately after the marathon to 0.59 mg/L (95% CI: 0.08–1.10) (Figure 7). Heterogeneity of the results was considerable for both measurements, with I^2^ values of 96% and 95%, respectively, both statistically significant.

### 3.3. Other Biomarkers

The mean urinary creatinine level before the marathon was 134.28 mg/dL (95% CI: 4.9–263.66) (Supplementary Table S2). It nearly doubled immediately after the marathon, reaching 283.09 mg/dL (95% CI: 127.91–438.27) (Supplementary Table S3), and then decreased at 24 h post-marathon to 103.74 mg/dL (95% CI: 79.5–127.97), falling below baseline levels (Supplementary Table S4).

The mean blood urea nitrogen-to-creatinine (BUN/Cr) ratio was 17.33 (95% CI: 15.68–18.98) before the marathon (Supplementary Table S2), decreased slightly to 15.33 (95% CI: 13.72–16.95) immediately after the marathon (Supplementary Table S3), and rose modestly above baseline to 19.33 (95% CI: 16.75–21.92) at 24 h post-marathon (Supplementary Table S4).

The mean serum urea level before the marathon was 35.2 mg/dL (95% CI: 31.2–39.2) (Supplementary Table S2), and it increased immediately after the marathon to 43.2 mg/dL (95% CI: 38.97–47.43) (Supplementary Table S3).

The mean value of TIMP-2 before the marathon was 2.4 ng/mL (95% CI: 2.15–2.65) (Supplementary Table S2). It rose markedly—by approximately fivefold—immediately after the marathon to 12.4 ng/mL (95% CI: 10.39–14.41) (Supplementary Table S3), and then decreased at 24 h post-marathon to 3.2 ng/mL (95% CI: 2.26–4.14), approaching baseline levels (Supplementary Table S4).

The mean value of IGFBP-7 before the marathon was 32.3 ng/mL (95% CI: 27.06–37.54) (Supplementary Table S2). It increased substantially—approximately sevenfold—immediately after the marathon to 236.6 ng/mL (95% CI: 188.33–284.87) (Supplementary Table S3), and decreased at 24 h post-marathon to 36.5 ng/mL (95% CI: 21.27–51.73), returning close to baseline levels (Supplementary Table S4).

The mean value of the TIMP-2*IGFBP-7 product before the marathon was 0.1 (95% CI: 0.08–0.12) (Supplementary Table S2). It increased dramatically—by 47 times—immediately after the marathon to 4.74 (95% CI: 2.92–6.56) (Supplementary Table S3), then decreased to 0.18 (95% CI: 0.07–0.29) at 24 h post-marathon, remaining nearly double the baseline value (Supplementary Table S4).

The mean urinary L-FABP (liver-type fatty acid binding protein) value before the marathon was 0.23 ng/mL (95% CI: 0.11–0.34) (Supplementary Table S2). It increased significantly—approximately twelve-fold—immediately after the marathon to 2.76 ng/mL (95% CI: 2.06–3.46) (Supplementary Table S3), then decreased at 24 h post-marathon to 0.63 ng/mL (95% CI: 0.38–0.88), roughly three times the baseline level (Supplementary Table S4).

The mean plasma NGAL value before the marathon was 43 ng/mL (95% CI: –25.75 to 111.76) (Supplementary Table S2). It doubled immediately after the marathon, reaching 100.68 ng/mL (95% CI: –5.57 to 206.94) (Supplementary Table S3), and then dropped substantially at 24 h post-marathon to 10.7 ng/mL (95% CI: 7.8–13.6), approximately one-quarter of the initial value (Supplementary Table S4).

The mean plasma KIM-1 value before the marathon was 1123.44 pg/mL (95% CI: –944.39 to 3191.26) (Supplementary Table S2). It increased immediately after the marathon to 1682.08 pg/mL (95% CI: –1453.4 to 4817.57) (Supplementary Table S3), and rose further at 24 h post-marathon to 2700 pg/mL (95% CI: 2072.81–3327.19), approximately double the baseline value (Supplementary Table S4).

The mean copeptin level before the marathon was 3.08 pmol/L (95% CI: 2.84–3.33) (Supplementary Table S2). It increased dramatically—about 15-fold—immediately after the marathon to 47.53 pmol/L (95% CI: 24.86–70.2) (Supplementary Table S3), and then decreased at 24 h post-marathon to 4.44 pmol/L (95% CI: 3.05–5.83), approaching the initial level (Supplementary Table S4).

The mean plasma TNF-alpha level before the marathon was 1.76 pg/mL (95% CI: 1.58–1.94) (Supplementary Table S2), and it increased after the marathon to 2.7 pg/mL (95% CI: 2.43–2.97) (Supplementary Table S3).

The mean urinary TNF-alpha value before the marathon was 0.02 pg/mL (95% CI: 0.01–0.03) (Supplementary Table S2). It increased fivefold immediately after the marathon to 0.1 pg/mL (95% CI: 0.05–0.15) (Supplementary Table S3), then decreased at 24 h post-marathon to 0.02 pg/mL (95% CI: 0.01–0.03), returning to baseline levels (Supplementary Table S4).

The mean plasma MCP-1 level before the marathon was 153.41 pg/mL (95% CI: 142.44–164.38) (Supplementary Table S2), and it doubled immediately after the marathon to 392.31 pg/mL (95% CI: 335.95–448.66) (Supplementary Table S3).

The mean urinary MCP-1 level before the marathon was 53.03 pg/mL (95% CI: 38.05–68.00) (Supplementary Table S2). It increased substantially—approximately tenfold—immediately after the marathon to 574.3 pg/mL (95% CI: 79.02–1069.59) (Supplementary Table S3), and then decreased at 24 h post-marathon to 202.98 pg/mL (95% CI: 100.04–305.91), remaining about four times higher than the baseline value (Supplementary Table S4).

The mean plasma YKL-40 level before the marathon was 29.33 ng/mL (95% CI: 23.04–35.63) (Supplementary Table S2), and it increased immediately after the marathon to 40.04 ng/mL (95% CI: 34.19–45.9) (Supplementary Table S3).

The mean urinary YKL-40 level before the marathon was 282.66 pg/mL (95% CI: –114.39 to 679.71) (Supplementary Table S2). It increased markedly—approximately fivefold—immediately after the marathon to 1530.34 pg/mL (95% CI: 361.32–2699.36) (Supplementary Table S3), and then decreased at 24 h post-marathon to 219.23 pg/mL (95% CI: 105.68–332.78), slightly below the initial value (Supplementary Table S4).

### 3.4. Identification of Biomarker Trends

**Figure 8:**
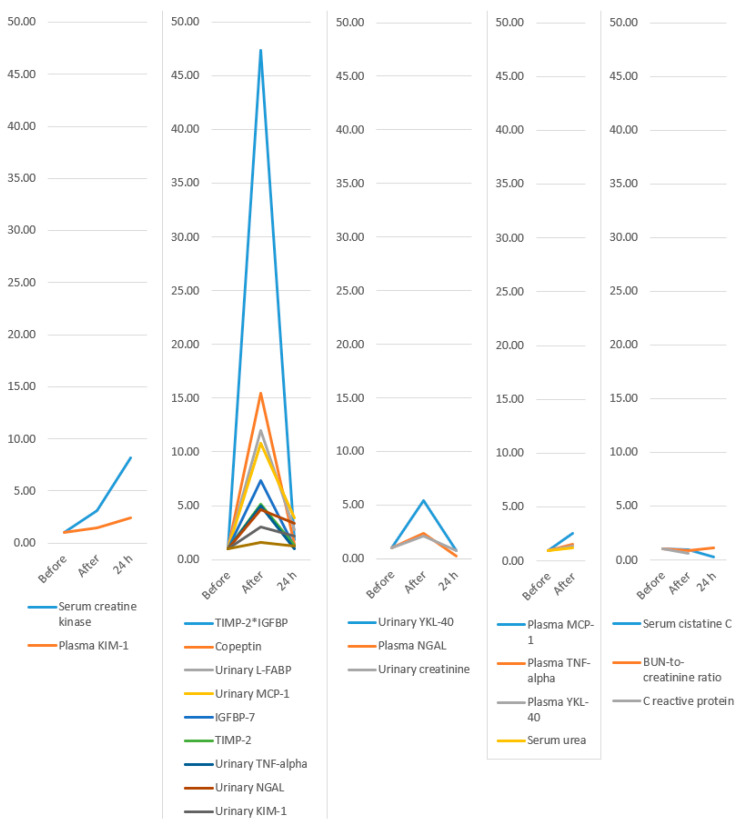
Evolution of biomarkers: only increase; increase and decrease but remain above baseline; increase and decrease below baseline; increase with no 24 h values available; and decrease below baseline after the marathon. KIM-1, Kidney Injury Molecule-1; TIMP-2, Tissue Inhibitor of Metalloproteinases-2; IGFBP-7, Insulin-like Growth Factor Binding Protein-7; L-FABP, Liver-type fatty acid binding protein; MCP-1, Monocyte chemoattractant protein-1; TNF-alpha, Tumor necrosis factor alpha; NGAL, Neutrophil Gelatinase-Associated Lipocalin; YKL-40, Chitinase 3-like protein 1; BUN, Blood Urea Nitrogen; *, represents the multiplication sign.

The temporal evolution of biomarker levels is illustrated in Figure 8 and detailed in Table 3.

### 3.5. Risk of Bias Assessment

We assessed the methodological quality of the selected articles (Table 4) with the quality assessment tool for before and after (pre–post) studies with no control group from the National Heart, Lung, and Blood Institute [21]. All the studies presented a clear question or study objective, and had eligible participants for the intervention (marathon) who were all enrolled in the study. All the studies had the marathon as an intervention, and only participants who finished it were included. The outcome measures were prespecified, clearly defined, valid, reliable, and assessed consistently across all study participants in all studies since there were biomarker measurements from blood, serum, plasma, or urine samples. The blinding of outcome measurements was not reported in any study, but being a laboratory measurement, there is no risk of bias. Only one study reported the sample size calculation, and that it was sufficient to provide confidence in the results, but it was performed after the data collection. Nevertheless, statistically significant results indicate that the sample size was enough for the purpose. The loss to follow-up after baseline was 20% or less in six studies, in two studies it was above 20%, and one study did not report this information. All but one study (88.9%) presented correct statistical methods for pair-wise comparisons between repeated measurements. Nevertheless, our review could not include the comparison for repeated measurements since the data for this was not reported (e.g., stating only that the *p*-value was less than a limit, without reporting the actual *p*-value, or confidence interval, or standard deviation of the change), thus the choice of test did not impact our results. All the studies used multiple measurements: at baseline, immediately after the marathon, and 24 h, or later, after the marathon. The intervention was conducted at a group level, but the statistical analysis considered the use of individual-level data, which is appropriate. Seven (77.8%) clearly presented the selection criteria. Four supplementary questions were assessed in addition to those in the NHLBI assessment tool. Only one study used clear exclusion criteria for eating food or alcohol consumption before the baseline measurement. Seven (77.8%) studies clearly excluded comorbidities that could have altered the measurements. Four studies (44.4%) clearly indicated the exclusion of participants who took medication (e.g., anti-inflammatory drugs) that could have altered the measurements. Only two studies (22.2%) presented the use of water, electrolytes, and food during the marathon.

## 4. Discussion

Our systematic review and meta-analysis succeeded in achieving its objectives. Most biomarkers showed an increase in value immediately after the marathon compared to pre-marathon levels. The largest increases (as a multiple of the baseline value) were observed for TIMP-2*IGFBP (47-fold), copeptin (15-fold), urinary L-FABP (12-fold), urinary MCP-1 (10-fold), IGFBP-7 (7-fold), urinary YKL-40 (5-fold), and TIMP-2 (5-fold). These may represent the most promising biomarker candidates for future research and potential clinical application. Several patterns of biomarker evolution were observed: some increased without decreasing even at 24 h after the marathon (serum creatine kinase and plasma KIM-1); others increased post-marathon and decreased at 24 h while remaining above baseline (TIMP-2IGFBP, copeptin, urinary L-FABP, urinary MCP-1, IGFBP-7, TIMP-2, urinary TNF-alpha, urinary NGAL, urinary KIM-1, and serum creatinine); some increased after the marathon and then fell below baseline at 24 h (urinary YKL-40, plasma NGAL, and urinary creatinine); a few increased post-marathon with no available 24 h data (plasma MCP-1, plasma TNF-alpha, plasma YKL-40, and serum urea); and others decreased immediately post-marathon below baseline (serum cystatin C, which decreased further at 24 h; BUN-to-creatinine ratio, which increased above baseline at 24 h; and C-reactive protein, for which no 24 h values were available).

The data provided by the studies did not allow for statistical testing to determine whether there were significant differences before and after the marathon. Most studies reported statistical test results, which were statistically significant for most of the evaluated biomarkers. Even though change data were not available for meta-analysis, the magnitude of the observed changes from baseline suggests that a meta-analysis including such data would have likely yielded statistically significant results.

This is the first systematic review with meta-analysis focusing on AKI biomarkers. Thus, it adds a quantitative perspective on the topic, coupled with a bird’s-eye view comparison of the most important increases in biomarkers in AKI. We standardized the evolution of biomarkers to be able to make comparisons between them possible. We found that some of them had very important increases immediately after the marathon. Increases over tenfold were observed for TIMP-2*IGFBP, copeptin, uL-FABP, and uMCP-1. Increases over 5-fold were observed for IGFBP-7, uTNF-alpha, and uYKL-40. Increases over 2.5-fold were observed for sCrK, uNGAL, uKIM-1, and pMCP-1. The others had lower increases: pKIM-1, sCr, pNGAL, uCr, pTNF-alpha, pYKL-40, and serum urea. Lastly, there were some biomarkers that diminished after the marathon: sCys C, BUN/Creatinine ratio, and C-reactive protein.

During the marathon, the intense exercise has several effects. One is the reduction in blood flow in the kidney [27] that can induce ischemia, which comes with a plethora of modified biomarkers. Dehydration is another factor that can induce ischemia. Another effect is the increase in the core temperature during running [28]. A quasi-randomized controlled trial showed how increased core temperature and dehydration increase the risk of AKI [29]. ATP depletion is a hypothesized mechanism of AKI [30].

TIMP-2*IGFBP was the most sensitive biomarker of the ones observed. Tissue inhibitor of metal proteinase 2 is a member of a family of proteins, named the TIMP family [31]. These proteins include matrix metalloproteinases, which are peptidases. TIMP-2 is an important modulator of extracellular matrix turnover and contributes to several physiological and pathological processes, including kidney damage and healing [32]. Although IGFBP-7 is part of the insulin-like growth factor (IGF) binding protein family, it has roles in addition to not being related to IGF. It causes the inhibition of cell growth and cell apoptosis by activating a specific pathway (ERK1/2) [33]. The combination of the two biomarkers known as TIMP-2*IGFBP, and its assessment as a diagnostic utility was first presented by Kashani et al. 2013 [34].

Copeptin had the second most important change, and only one study assessed its evolution [8]. Copeptin is a peptide used as a marker of vasopressin secretion. It is used for diagnosis and prognosis of different diseases, including renal disease [35]

L-FABP had the third most important change immediately after the marathon. It is produced in renal proximal tubular cells and released into the urine in reaction to tubular hypoxia [36]. In addition to its renal secretion, L-FABP can be produced by the liver, too. Studies have shown that the urinary L-FABP can be used as a marker of kidney injury, specifically since it is independent of the serum values due to its liver production [37]

MCP-1 had the fourth most important change immediately after the marathon. Monocyte chemoattractant protein-1 belongs to the CC chemokine family. It plays an important role in the inflammatory process by drawing in or promoting the expression of other inflammatory cells and molecules [38]. MCP-1 has been shown to be associated with a rise in renal monocyte infiltration [39]. After renal ischemia–reperfusion damage, MCP-1 prevents the kidney from the immediate inflammatory response [40].

TNF alpha belongs to the TNF family that includes TNF alpha, beta, CD40 ligand, and others [41]. All these are cytokines with roles in inflammation, apoptosis, and tumor lysis. Macrophages are the main source of TNF alpha, though other cells can produce it too. TNF alpha is implicated in numerous functions: inducing fever, apoptosis, necrosis, tumor genesis, progression, and systemic inflammatory reactions [41]. Higher levels of urinary TNF alpha were observed in participants with acute interstitial nephritis [42]. Also, polymorphisms of TNF alpha were seen as predictive of acute kidney injury [43].

YKL-40 is a glycoprotein from the chitinase protein family [44], although it lacks chitinase activity [45]. It is secreted by many cell types, including macrophages, neutrophils, vascular smooth muscle cells, and chondrocytes [45]. It is implicated in many inflammatory diseases, including infections, cardiovascular, neurologic, respiratory, gastrointestinal, urinary, musculoskeletal, immunological, and endocrinological diseases [46]. YKL-40 levels increase after kidney injury (e.g., after transplantation) [47].

Two studies were performed only on male participants, and in two others, the percentage of female participants was 18% and 29%, respectively. In five (more than half) of the included studies, the proportions of male and female participants were approximately equal (with a slightly higher prevalence in females). Thus, some studies had male prevalence, and others were similarly represented. Nevertheless, we recognize that there are differences in the function of sex concerning physiology. These can influence the levels and the evolution in time of certain biomarkers based on sex. Women usually have lower serum creatinine levels [48] due to less muscle volume. Also, the sex hormones may influence the renal responses to exercise. Despite these differences, the mechanisms of exercise-induced kidney stress, such as transient hypoperfusion, dehydration, and oxidative stress, are likely present in both sexes.

### 4.1. Limitations

Statistical heterogeneity of the results was substantial, or considerable, and statistically significant in most of the analyses where it could be calculated. This can be explained by clinical heterogeneity among the included studies: differences in participants’ age, sex distribution, genetic predisposition, prior participation in marathon events, previous health condition, athlete type (professional or recreational), training volume and frequency (weekly running distance and duration), possible comorbidities that were not excluded, use of anti-inflammatory medications (e.g., NSAID), fluid (hydration status), electrolytes and food intake (before and during the marathon), environmental temperature during the event, and course difficulty (flat vs. uphill or downhill terrain).

None of the included studies performed adjusted analyses to account for potential confounding variables, such as those mentioned above as sources of variability. This may introduce a risk of confounding. Biomarker measurements were conducted using appropriate techniques, so measurement error at the sample level is unlikely. The laboratory measurements were not clearly reported, if they were blinded. Nevertheless, by being objective assessments, the risk of bias is avoided. However, the timing of measurement was inconsistent, particularly for the pre-marathon assessment. In some studies, baseline measurements were taken on the day of the marathon, while in others, they were obtained in the days, or even weeks, before. Of course, the participants were most likely healthy individuals, and we would expect limited variability in biomarker levels over time, reducing the likelihood of significant bias.

Some biomarkers, such as copeptin and IGFBP-7, were assessed in only one or two studies in our review. Thus, while their change post-marathon was important, the confidence in the results remains cautious. These findings should be viewed as exploratory and sources of hypotheses and not confirmatory.

### 4.2. Study Strengths

This study provides valuable insights into the early diagnosis of acute kidney injury in marathon runners using a wide range of biomarkers. These findings may support the role of the studied biomarkers in AKI. Moreover, it shows the comparative evolution of biomarkers during and after a marathon run. This can suggest which one of the biomarkers might be more sensitive to identifying AKI.

Currently, the use of kidney injury biomarkers in marathon runners is only an area of research. Future integration of these biomarkers for real-time or field screening of marathon runners for AKI would come with some challenges: costs of the analyses, the use of laboratories, the time to do the analysis and to obtain the result, and the availability of the marathon runners to be evaluated. For some biomarkers, such as urinary NGAL [49] or TIMP-2*IGFBP-7 [50], rapid tests were developed that are now used in some critical care units. These might be more feasible to be used in the framework of a marathon race at the end, and not in real-time, since the test is based on urine samples and requires 10–20 min to provide a result. Since most of the biomarkers return to their baseline values, the necessity of performing the tests on all marathon runners is limited. A pragmatic approach could be the identification of participants at high risk for AKI based on a questionnaire screening before the marathon, and performing these tests on this subgroup. If future portable tests were developed, then they could be used in real-time for high-risk participants.

Future research could focus on evaluating the long-term effects of repeated marathon participation. Expanding the study to include other types of endurance events and a larger sample size could offer a more comprehensive understanding of the impact of intense physical exertion on kidney function. Including other markers, such as oxidative stress and antioxidant protection enzymes, might be another venue of study. Animal studies can provide additional information in a controlled setting to complete the picture of evidence obtained in human studies.

## 5. Conclusions

The study showed significant increases in most biomarkers immediately after the marathon compared to baseline values. The largest increases were observed in TIMP-2*IGFBP, copeptin, urinary L-FABP, urinary MCP-1, IGFBP-7, urinary YKL-40, and TIMP-2, suggesting that these biomarkers are promising candidates for future research. Several patterns of biomarker evolution were observed: some increased without decreasing even at 24 h after the marathon (serum creatine kinase and plasma KIM-1); others increased post-marathon and decreased at 24 h while remaining above baseline (TIMP-2IGFBP, copeptin, urinary L-FABP, urinary MCP-1, IGFBP-7, TIMP-2, urinary TNF-alpha, urinary NGAL, urinary KIM-1, and serum creatinine); and some increased after the marathon and then fell below baseline at 24 h (urinary YKL-40, plasma NGAL, and urinary creatinine).

## Figures and Tables

**Table 1:** Characteristics of the studies included in the review.

Study	Country	Region	Study Design	Marathon Place	Mean Age (Years)	Females (%)	BMI (kg/m^2^)
Leckie, 2023 [20]	United Kingdom	Europe	prospective cohort	Brighton 2019	41 ± 10	18	NR
Kosaki, 2022 [21]	Japan	Europe	prospective cohort	Tsukuba 2018	23 ± 1	0	21.3 ± 1.5
Atkins, 2021 [22]	USA	America	prospective cohort	Boston 2019	46 ± 10	49	
Nescolarde, 2020 [23]	Spain	Europe	prospective cohort	Barcelona 2017	41 ± 4	0	24.0 ± 2.1
Mansour, 2019 [8]	USA	America	prospective cohort	Hartford 2017	37 (35–44)	57	24 (22–25)
Mansour, 2017 [7]	USA	America	prospective cohort	Hartford 2015	44.2 ± 12.9	59	22.4 ± 2.4
McCullough, 2011 [24]	USA	America	prospective cohort	Detroit 2008	38.7 ± 9.0	52	23.0 ± 2.6
Bekos, 2016 [25]	Austria	Europe	prospective cohort	Vienna 2012	36.83 ± 7.56	29	22.29 ± 2.16
Hewing, 2015 [26]	Germany	Europe	prospective cohort	Berlin 2006, 2007	50.3 [22–72]	53	22.4 ± 2.1

Data are presented as mean ± standard deviation or median (interquartile range) [minimum–maximum]; NR, not reported; BMI, body mass index.

**Table 2:** Characteristics of the studies included in the review (continued).

Study	Previous Marathons (Number)	km/Week	Running History (Years)	AKI Definition Criteria	AKI Stage I *	Measured Parameters
Leckie, 2023 [20]	5 ± 7 [0–39]	43 ± 17 [15–72]	NR	KDIGO	Yes	sCr, uCr, TIMP-2, IGFBP-7, TIMP-2*IGFBP-7
Kosaki, 2022 [21]	NR	NR	NR	AKIN	Yes	sCr, L-FABP
Atkins, 2021 [22]	NR	NR	NR	NR	No	sCr, uCr, sCys C, uNGAL
Nescolarde, 2020 [23]	NR	NR	8.2 ± 5.1	AKIN	Yes	sCr, sCK, Uree serică, sPCR
Mansour, 2019 [8]	3 (1–9)	47 (27–58)	9 (5, 12)	AKIN	Yes	sCr, BUN/Cr, sCK, uNGAL, pNGAL, uKIM-1, pKIM-1, copeptină, TNF-alpha plasmatic, MCP-1 plasmatic, MCP-1 urinar, YKL-40 plasmatic, YKL-40 urinar
Mansour, 2017 [7]	5 (2–16)	51 ± 16	12.0 (5.0–15.0)	AKIN	Yes	sCr, sCK, uNGAL, uKIM-1, TNF-alpha urinar, MCP-1 urinar, YKL-40 urinar
McCullough, 2011 [24]	2.3 ± 3.0	27 ± 19		AKIN	Yes	sCr, sCys C, pNGAL, pKIM-1, sCK
Bekos, 2016 [25]	NR	61.85 ± 20.58	NR	AKIN	Yes	sCK, sCRP
Hewing, 2015 [26]	6.0 [3.0–13.0]	50.0 [40.0–65.0]	10.0 [6.0–20.0]	RIFLE	Yes	sCr, sCys C, sCRP

Data are presented as mean ± standard deviation or median (interquartile range) [minimum–maximum]; NR, not reported; AKI, acute kidney injury; KDIGO, Kidney Disease: Improving Global Outcomes; AKIN, Acute Kidney Injury Network; RIFLE, Risk, Injury, Failure, Loss, and End-stage Kidney Disease; sCr, serum creatinine; uCr, urinary creatinine; TIMP-2, Tissue Inhibitor of Metalloproteinases-2; IGFBP-7, Insulin-like Growth Factor Binding Protein-7; L-FABP, liver-type fatty acid binding protein; sCK, serum creatine kinase; sCys C, serum cystatin C; uNGAL, urinary Neutrophil Gelatinase-Associated Lipocalin; BUN/Cr, blood urea nitrogen/creatinine ratio; pNGAL, plasma Neutrophil Gelatinase-Associated Lipocalin; uKIM-1, urinary Kidney Injury Molecule-1; pKIM-1, plasma Kidney Injury Molecule-1; sCRP, serum C-reactive protein; TNF-alpha, tumor necrosis factor alpha; YKL-40, chitinase 3-like protein 1; MCP-1, monocyte chemoattractant protein-1; *, studies that reported the number of subjects with stage 1 AKI.

**Table 3:** Evolution of biomarkers expressed as a multiple of the pre-marathon value.

Pattern of Change	Biomarker	Pre-Marathon	Post-Marathon	24 h
Increase post-marathon, further increase at 24 h	Serum creatine kinase	1.00	3.08	8.16
	Plasma KIM-1	1.00	1.50	2.40
Increase post-marathon, decrease at 24 h above baseline	TIMP-2*IGFBP	1.00	47.40	1.80
	Copeptin	1.00	15.43	1.44
	Urinary L-FABP	1.00	12.00	2.74
	Urinary MCP-1	1.00	10.83	3.83
	IGFBP-7	1.00	7.33	1.13
	TIMP-2	1.00	5.17	1.33
	Urinary TNF-alpha	1.00	5.00	1.00
	Urinary NGAL	1.00	4.63	3.32
	Urinary KIM-1	1.00	2.99	2.16
	Serum creatinine	1.00	1.57	1.27
Increase post-marathon, decrease at 24 h below baseline	Urinary YKL-40	1.00	5.41	0.78
	Plasma NGAL	1.00	2.34	0.25
	Urinary creatinine	1.00	2.11	0.77
Increase post-marathon (no 24 h values)	Plasma MCP-1	1.00	2.56	–
	Plasma TNF-alpha	1.00	1.53	–
	Plasma YKL-40	1.00	1.37	–
	Serum urea	1.00	1.23	–
Decrease post-marathon	Serum cystatin C	1.00	0.92	0.26
	BUN/Creatinine ratio	1.00	0.88	1.12
	C-reactive protein	1.00	0.63	–

KIM-1, Kidney Injury Molecule-1; TIMP-2, Tissue Inhibitor of Metalloproteinases-2; IGFBP-7, Insulin-like Growth Factor Binding Protein-7; MCP-1, Monocyte chemoattractant protein-1; TNF-alpha, Tumor necrosis factor alpha; NGAL, Neutrophil Gelatinase-Associated Lipocalin; YKL-40, Chitinase 3-like protein 1; BUN, Blood Urea Nitrogen; *, represents the multiplication sign.

**Table 4:** Risk of bias assessment using the quality assessment tool for before and after (pre–post) studies with no control group from the National Heart, Lung, and Blood Institute (NHLBI).

Criteria/Study	Leckie, 2023 [20]	Kosaki, 2022 [21]	Atkins, 2021 [22]	Nescolarde, 2020 [23]	Mansour, 2019 [8]	Mansour, 2017 [7]	McCullough, 2011 [24]	Bekos, 2016 [25]	Hewing, 2015 [26]
1. Was the study question or objective clearly stated?	Yes	Yes	Yes	Yes	Yes	Yes	Yes	Yes	Yes
2. Were eligibility/selection criteria for the study population prespecified and clearly described?	No	No	Yes	Yes	Yes	Yes	Yes	Yes	Yes
3. Were the participants in the study representative of those who would be eligible for the test/service/intervention in the general or clinical population of interest?	Yes	Yes	Yes	Yes	Yes	Yes	Yes	Yes	Yes
4. Were all eligible participants who met the prespecified entry criteria enrolled?	Yes	Yes	Yes	Yes	Yes	Yes	Yes	Yes	Yes
5. Was the sample size sufficiently large to provide confidence in the findings?	NR	NR	NR	NR	NR	NR	Yes	NR	NR
6. Was the test/service/intervention clearly described and delivered consistently across the study population?	Yes	Yes	Yes	Yes	Yes	Yes	Yes	Yes	Yes
7. Were the outcome measures prespecified, clearly defined, valid, reliable, and assessed consistently across all study participants?	Yes	Yes	Yes	Yes	Yes	Yes	Yes	Yes	Yes
8. Were the people assessing the outcomes blinded to the participants’ exposures/interventions?	NR	NR	NR	NR	NR	NR	NR	NR	NR
9. Was the loss to follow-up after baseline 20% or less? Post-marathon/24 h after. Were those lost to follow-up accounted for in the analysis?	Yes/No	NR	Yes/Yes	Yes/Yes	Yes/Yes	Yes/Yes	Yes/Yes	No/No	Yes/Yes
10. Did the statistical methods examine changes in outcome measures from before to after the intervention? Were statistical tests performed that provided *p*-values for the pre-to-post changes?	Yes	Yes	Yes	Yes	Yes	Yes	Yes	No	Yes
11. Were outcome measures of interest taken multiple times before the intervention and multiple times after the intervention (i.e., did they use an interrupted time-series design)?	Yes	Yes	Yes	Yes	Yes	Yes	Yes	Yes	Yes
12. If the intervention was conducted at a group level (e.g., a whole hospital, a community, etc.), did the statistical analysis take into account the use of individual-level data to determine effects at the group level?	Yes	Yes	Yes	Yes	Yes	Yes	Yes	Yes	Yes
13. * Clear exclusion criteria for: alcohol and food abstinence 12 h before baseline measurement	NR	Yes	NR	NR	NR	NR	NR	NR	NR
14: * Exclusion of comorbidities that could alter measurements	NR	NR	Yes	Yes	Yes	Yes	Yes	Yes	Yes
15: * Exclusion of medication that could alter measurements	NR	NR	Yes	NR	Yes	Yes	NR	Yes	NR
16: * Presenting the use of water, electrolytes, and food during the marathon	NR	NR	NR	Yes	NR	NR	Yes	NR	NR

*, supplementary questions, added by the authors of this paper, different from the NHLBI questions; NR, not reported; NA, not applicable.

## Data Availability

Data is contained within the paper.

## Supplementary Materials

The following supporting information can be downloaded at: https://www.mdpi.com/article/10.3390/medicina61101775/s1. Supplementary Table S1: Search strategy in bibliographic databases; Supplementary Table S2: Results of the meta-analyses of mean biomarker values before the marathon, including the inconsistency index (I^2^), associated *p*-value, Egger’s test for publication bias, and the number of studies included for each biomarker; Supplementary Table S3: Results of the meta-analyses of mean biomarker values immediately after the marathon, including the inconsistency index (I^2^), associated *p*-value, Egger’s test for publication bias, and the number of studies included for each biomarker; Supplementary Table S4: Results of the meta-analyses of mean biomarker values 24 h after the marathon, including the inconsistency index (I^2^), associated *p*-value, Egger’s test for publication bias, and the number of studies included for each biomarker.

## Abbreviations

The following abbreviations are used in this manuscript:

Abbreviation	Explanation
AKI	Acute Kidney Injury
AKIN	Acute Kidney Injury Network
BUN	Blood Urea Nitrogen
BUN/Cr	Blood Urea Nitrogen/Creatinine Ratio
BMI	Body Mass Index
CI	Confidence Interval
CRP	C-Reactive Protein
IGFBP-7	Insulin-like Growth Factor Binding Protein-7
KDIGO	Kidney Disease: Improving Global Outcomes
KIM-1	Kidney Injury Molecule-1
L-FABP	Liver-type Fatty Acid Binding Protein
MCP-1	Monocyte Chemoattractant Protein-1
NA	Not Applicable
NGAL	Neutrophil Gelatinase-Associated Lipocalin
NHLBI	National Heart, Lung, and Blood Institute
NR	Not Reported
pKIM-1	Plasma Kidney Injury Molecule-1
pNGAL	Plasma Neutrophil Gelatinase-Associated Lipocalin
RIFLE	Risk, Injury, Failure, Loss, and End-Stage Kidney Disease
sCK	Serum Creatine Kinase
sCRP	Serum C-Reactive Protein
sCr	Serum Creatinine
sCys C	Serum Cystatin C
TIMP-2	Tissue Inhibitor of Metalloproteinases-2
TNF-α	Tumor Necrosis Factor Alpha
uCr	Urinary Creatinine
uKIM-1	Urinary Kidney Injury Molecule-1
uNGAL	Urinary Neutrophil Gelatinase-Associated Lipocalin
YKL-40	Chitinase 3-like Protein 1
