# Supplementary material for: Acute Kidney Injury Biomarkers in Marathon Runners: Systematic Review and Meta-Analysis
Source: Medicina (Kaunas). 2025 Oct 1;61(10):1775. doi: 10.3390/medicina61101775 (PMC12566084; doi:10.3390/medicina61101775)
Supplement: Supplementary file 1 [file medicina-61-01775-s001.zip › medicina-3857799-supplementary.pdf]

Search strategy

Supplementary Table S1. Search strategy in bibliographic databases.

**PubMed**
(<br>("cystatin c"[MeSH Terms] OR "cystatin c"[All Fields] OR "CYS-C"[All Fields] OR "CYS C"[All Fields]) OR<br>("lipocalin 2"[MeSH Terms] OR "lipocalin 2"[All Fields] OR ("neutrophil"[All Fields] AND "gelatinase"[All Fields] AND "associated"[All Fields] AND "lipocalin"[All Fields]) OR "neutrophil gelatinase associated lipocalin"[All Fields] OR "NGAL"[All Fields]) OR<br>((("kidney"[MeSH Terms] OR "kidney"[All Fields] OR "kidneys"[All Fields]) AND ("injury"[All Fields] "injurie"[All Fields] OR "injuries"[MeSH Subheading] OR "injuries"[All Fields]) AND "molecule-1"[All Fields]) OR ("kidney injury molecule-1"[All Fields]) OR ("kidney injury molecule 1"[All Fields]) OR "KIM1"[All Fields] OR "KIM-1"[All Fields]) OR<br>("creatine kinase"[MeSH Terms] OR ("creatine"[All Fields] AND "kinase"[All Fields]) OR "creatine kinase"[All Fields]) OR<br>(("serum"[MeSH Terms] OR "serum"[All Fields]) AND ("creatinin"[All Fields] OR "creatinine"[MeSH Terms] OR "creatinine"[All Fields] OR "creatinines"[All Fields])) OR<br>(("Insulin-like"[All Fields] AND ("factor"[All Fields] OR "factors"[All Fields]) AND ("carrier proteins"[MeSH Terms] OR ("carrier"[All Fields] AND "proteins"[All Fields]) OR "carrier proteins"[All Fields] OR ("binding"[All Fields] AND "protein"[All Fields]) OR "binding protein"[All Fields]) AND "7"[All Fields]) OR "Insulin-like growth factor binding protein 7"[All Fields] OR "Insulin-like growth factor binding protein-7"[All Fields] OR "IGFBP7"[All Fields] OR "IGFBP-7"[All Fields]) OR<br>((("tissues"[MeSH Terms] OR "tissues"[All Fields] OR "tissue"[All Fields]) AND ("antagonists and inhibitors"[MeSH Subheading] OR ("antagonists"[All Fields] AND "inhibitors"[All Fields]) OR "antagonists and inhibitors"[All Fields] OR "inhibitors"[All Fields] OR "inhibitor"[All Fields] OR "inhibitor s"[All Fields]) AND "metalloproteinases-2"[All Fields]) OR "tissue inhibitor of metalloproteinases-2"[All Fields] OR "tissue inhibitor of metalloproteinases 2"[All Fields] OR "TIMP2"[All Fields] OR "TIMP-2"[All Fields]) OR

("fatty acid binding proteins"[MeSH Terms] OR ("fatty"[All Fields] AND "acid binding"[All Fields] AND "proteins"[All Fields]) OR "fatty acid binding proteins"[All Fields] OR ("liver"[All Fields] AND "fatty"[All Fields] AND "acid"[All Fields] AND "binding"[All Fields] AND "protein"[All Fields]) OR "liver fatty acid binding protein"[All Fields] OR "liver-type fatty acid binding protein"[All Fields] OR "L-FABP"[All Fields]) OR<br>("biomarkers"[MeSH Terms] OR "biomarkers"[All Fields] OR "biomarker"[All Fields])<br>) AND<br>("acute kidney injury"[MeSH Terms] OR ("acute"[All Fields] AND "kidney"[All Fields] AND "injury"[All Fields]) OR "acute kidney injury"[All Fields]) AND ("marathon running"[MeSH Terms] OR ("marathon"[All Fields] AND "running"[All Fields]) OR "marathon running"[All Fields] OR "marathon"[All Fields] OR "marathons"[All Fields] OR "marathoner"[All Fields] OR "marathoners"[All Fields])
**EMBASE**
(<br>('cystatin c'/exp OR 'cystatin c' OR 'cys-c' OR 'cys c') OR<br>('lipocalin 2'/exp OR 'lipocalin 2' OR (('neutrophil'/exp OR 'neutrophil') AND ('gelatinase'/exp OR 'gelatinase') AND 'associated' AND ('lipocalin'/exp OR 'lipocalin'))) OR ('neutrophil gelatinase associated lipocalin'/exp OR 'neutrophil gelatinase associated lipocalin' OR 'ngal'/exp OR 'ngal') OR<br>((('kidney'/exp OR 'kidney' OR 'kidneys'/exp OR 'kidneys') AND (('injury'/exp OR 'injury') AND 'injurie' OR 'injuries'/exp OR 'injuries') AND 'molecule-1') OR 'kidney injury molecule-1'/exp OR 'kidney injury molecule-1' OR 'kidney injury molecule 1'/exp OR 'kidney injury molecule 1' OR 'kim1' OR 'kim-1') OR<br>((('creatine'/exp OR 'creatine') AND ('kinase'/exp OR 'kinase')) OR 'creatine kinase'/exp OR 'creatine kinase') OR<br>((('serum'/exp OR 'serum') AND ('creatinin'/exp OR 'creatinin' OR 'creatinine'/exp OR 'creatinine' OR 'creatinines'))) OR<br>(('insulin-like' AND ('factor' OR 'factors') AND (('carrier'/exp OR 'carrier') AND ('proteins'/exp OR 'proteins') OR 'carrier proteins'/exp OR 'carrier proteins' OR (('binding'/exp OR 'binding') AND ('protein'/exp OR 'protein')) OR 'binding protein'/exp OR 'binding protein') AND '7') OR 'insulin-like

growth factor binding protein 7' OR 'insulin-like growth factor binding protein-7' OR 'igfbp7' OR 'igfbp-7') OR<br>((('tissues'/exp OR 'tissues' OR 'tissue'/exp OR 'tissue') AND ('antagonists' AND ('inhibitors'/exp OR 'inhibitors') OR 'antagonists and inhibitors'/exp OR 'antagonists and inhibitors' OR 'inhibitors'/exp OR 'inhibitors' OR 'inhibitor'/exp OR 'inhibitor' OR 'inhibitor s') AND 'metalloproteinases-2') OR 'tissue inhibitor of metalloproteinases-2' OR 'tissue inhibitor of metalloproteinases 2' OR 'timp2' OR 'timp-2'/exp OR 'timp-2') OR<br>(('fatty' AND 'acid binding' AND ('proteins'/exp OR 'proteins')) OR 'fatty acid binding proteins'/exp OR 'fatty acid binding proteins' OR (('liver'/exp OR 'liver') AND 'fatty' AND ('acid'/exp OR 'acid') AND ('binding'/exp OR 'binding') AND ('protein'/exp OR 'protein')) OR 'liver fatty acid binding protein'/exp OR 'liver fatty acid binding protein' OR 'liver-type fatty acid binding protein' OR 'l-fabp') OR<br>('biomarkers'/exp OR 'biomarkers' OR 'biomarker'/exp OR 'biomarker'))<br>AND ('acute' AND ('kidney'/exp OR 'kidney') AND ('injury'/exp OR 'injury') OR 'acute kidney injury'/exp OR 'acute kidney injury') AND<br>(('marathon'/exp OR 'marathon') AND ('running'/exp OR 'running') OR 'marathon running'/exp OR 'marathon running' OR 'marathon'/exp OR 'marathon' OR 'marathons' OR 'marathoner'/exp OR 'marathoner' OR 'marathoners'/exp OR 'marathoners')
**Web of Science**
TS=(<br>(<br>("cystatin c" OR "CYS-C" OR "CYS C") OR<br>("lipocalin 2" OR ("neutrophil" AND "gelatinase" AND "associated" AND "lipocalin") OR "neutrophil gelatinase associated lipocalin" OR "NGAL") OR<br>((("kidney" OR "kidneys") AND ("injury" OR "injuries") AND "molecule-1") OR ("kidney injury molecule-1") OR ("kidney injury molecule 1") OR "KIM1" OR "KIM-1") OR<br>("creatine kinase" OR ("creatine" AND "kinase")) OR<br>(("serum") AND ("creatinin" OR "creatinine" OR "creatinines")) OR<br>(("Insulin-like" AND ("factor" OR "factors") AND ("carrier proteins" OR ("carrier" AND "proteins") OR ("binding" AND "protein") OR "binding protein") AND "7") OR "Insulin-like growth factor binding protein 7" OR

"Insulin-like growth factor binding protein-7" OR "IGFBP7" OR "IGFBP-7")<br>OR<br>((("tissue" OR "tissues") AND ("antagonists and inhibitors" OR ("antagonists" AND "inhibitors") OR "inhibitors" OR "inhibitor" OR "inhibitor s") AND "metalloproteinases-2") OR "tissue inhibitor of metalloproteinases-2" OR "tissue inhibitor of metalloproteinases 2" OR "TIMP2" OR "TIMP-2")<br>OR<br>("fatty acid binding proteins" OR ("fatty" AND "acid binding" AND "proteins") OR ("liver" AND "fatty" AND "acid" AND "binding" AND "protein") OR "liver fatty acid binding protein" OR "liver-type fatty acid binding protein" OR "L-FABP") OR<br>("biomarkers" OR "biomarker")<br>) AND<br>("acute kidney injury" OR ("acute" AND "kidney" AND "injury")) AND<br>("marathon running" OR ("marathon" AND "running") OR "marathon" OR "marathons" OR "marathoner" OR "marathoners")<br>)
**LILACS**
tw:(<br>(<br>("cystatin c" OR "CYS-C" OR "CYS C") OR<br>("lipocalin 2" OR ("neutrophil" AND "gelatinase" AND "associated" AND "lipocalin") OR "neutrophil gelatinase associated lipocalin" OR "NGAL") OR<br>((("kidney" OR "kidneys") AND ("injury" OR "injuries") AND "molecule-1") OR ("kidney injury molecule-1") OR ("kidney injury molecule 1") OR "KIM1" OR "KIM-1") OR<br>("creatine kinase" OR ("creatine" AND "kinase")) OR<br>(("serum") AND ("creatinin" OR "creatinine" OR "creatinines")) OR<br>(("Insulin-like" AND ("factor" OR "factors") AND ("carrier proteins" OR ("carrier" AND "proteins") OR ("binding" AND "protein") OR "binding protein") AND "7") OR "Insulin-like growth factor binding protein 7" OR "Insulin-like growth factor binding protein-7" OR "IGFBP7" OR "IGFBP-7")<br>OR<br>((("tissue" OR "tissues") AND ("antagonists and inhibitors" OR ("antagonists" AND "inhibitors") OR "inhibitors" OR "inhibitor" OR "inhibitor s") AND "metalloproteinases-2") OR "tissue inhibitor of metalloproteinases-

2" OR "tissue inhibitor of metalloproteinases 2" OR "TIMP2" OR "TIMP-2")
OR
("fatty acid binding proteins" OR ("fatty" AND "acid binding" AND "proteins") OR ("liver" AND "fatty" AND "acid" AND "binding" AND "protein") OR "liver fatty acid binding protein" OR "liver-type fatty acid binding protein" OR "L-FABP") OR
("biomarkers" OR "biomarker")
) AND
("acute kidney injury" OR ("acute" AND "kidney" AND "injury")) AND
("marathon running" OR ("marathon" AND "running") OR "marathon" OR "marathons" OR "marathoner" OR "marathoners")
)

Meta-analysis detailed results

Supplementary Table S2. Results of the meta-analyses of mean biomarker values before the marathon, including the inconsistency index (I^2), associated p-value, Egger's test for publication bias, and the number of studies included for each biomarker.

Biomarker	Number studies	Mean (95% CI)	I^2 (95% CI)	p	Egger test	Studies
Serum creatinine (mg/dL)	7	0.88 (0.85 - 0.92)	84.7 (70.3 - 92.1)	< 0.001	0.533	McCullough, 2011; Hewing, 2015; Mansour, 2017; Mansour, 2019; Nescolarde, 2020; Kosaki, 2022; Leckie, 2023
Urinary creatinine (mg/dL)	2	134.28 (4.9 - 263.66)	NC		NC	Atkins, 2021; Leckie, 2023
BUN-to-creatinine ratio	1	17.33 (15.68 - 18.98)	NC		NC	Mansour, 2019
Serum urea (mg/dL)	1	35.2 (31.2 - 39.2)	NC		NC	Nescolarde, 2020
Serum creatine kinase (U/L)	5	138.31 (105.12 - 171.5)	74.9 (38 - 89.8)	0.003	0.607	McCullough, 2011; Bekos, 2016; Mansour, 2017; Mansour, 2019; Nescolarde, 2020
TIMP-2 (ng/dL)	1	2.4 (2.15 - 2.65)	NC		NC	Leckie, 2023
IGFBP-7 (ng/dL)	1	32.3 (27.06 - 37.54)	NC		NC	Leckie, 2023
TIMP-2*IGFBP $(ng/dL)^2/1000$	1	0.1 (0.08 - 0.12)	NC		NC	Leckie, 2023

Urinary L-FABP (µg/g creatinine)	1	0.23 (0.11 - 0.34)	NC		NC	Kosaki, 2022
Urinary NGAL (ng/mL)	4	8.92 (6.11 - 11.73)	47.4 (0 - 82.6)	0.127	0.327	McCullough, 2011; Mansour, 2017; Mansour, 2019; Atkins, 2021
Plasma NGAL (ng/mL)	2	43 (-25.75 - 111.76)	NC		NC	McCullough, 2011; Mansour, 2019
Serum cystatin C (mg/dL)	2	0.39 (-0.21 - 0.99)	NC		NC	McCullough, 2011; Hewing, 2015
Plasma KIM-1 (pg/mL)	2	1123.44 (-944.39 - 3191.26)	NC		NC	McCullough, 2011; Mansour, 2019
Urinary KIM-1 (pg/mL)	3	755.02 (-474.24 - 1984.29)	96.8 (93.4 - 98.4)	< 0.001	0.283	McCullough, 2011; Mansour, 2017; Mansour, 2019
Serum C-reactive protein (mg/L)	3	0.93 (0.03 - 1.82)	96.3 (92.2 - 98.2)	< 0.001	0.892	Hewing, 2015; Bekos, 2016; Nescolarde, 2020
Copeptin (pmol/L)	1	3.08 (2.84 - 3.33)	NC		NC	Mansour, 2019
Plasma TNF-alpha (pg/mL)	1	1.76 (1.58 - 1.94)	NC		NC	Mansour, 2019
Urinary TNF-alpha (pg/mL)	1	0.02 (0.01 - 0.03)	NC		NC	Mansour, 2017
Plasma MCP-1 (pg/mL)	1	153.41 (142.44 - 164.38)	NC		NC	Mansour, 2019
Urinary MCP-1 (pg/mL)	2	53.03 (38.05 - 68)	NC		NC	Mansour, 2017; Mansour, 2019
Plasma YKL-40 (ng/mL)	1	29.33 (23.04 - 35.63)	NC		NC	Mansour, 2019
Urinary YKL-40 (pg/mL)	2	282.66 (-114.39 - 679.71)	NC		NC	Mansour, 2017; Mansour, 2019

CI, confidence interval; NC, not calculable; BUN, Blood Urea Nitrogen; TIMP-2, Tissue Inhibitor of Metalloproteinases-2; IGFBP-7, Insulin-like Growth Factor Binding Protein-7; L-FABP, Liver-type fatty acid binding protein; NGAL, Neutrophil Gelatinase-Associated Lipocalin; KIM-1, Kidney Injury Molecule-1; TNF-alpha, Tumor necrosis factor alpha; MCP-1, Monocyte chemoattractant protein-1; YKL-40, Chitinase 3-like protein 1.

Supplementary Table S3. Results of the meta-analyses of mean biomarker values immediately after the marathon, including the inconsistency index (I^2), associated p-value, Egger's test for publication bias, and the number of studies included for each biomarker.

**Biomarker**	**Biomarker**	**Number studies**	**Mean (95% CI)**	**I^2 (95% CI)**	**p**	**Egger test**	**Studies**

Serum creatinine (mg/dL)	Creatinina serică (mg/dL)	8	1.38 (1.04 - 1.71)	97.7 (96.7 - 98.4)	< 0.001	0.081	McCullough, 2011; Hewing, 2015; Mansour, 2017; Mansour, 2019; Nescolarde, 2020; Atkins, 2021; Kosaki, 2022; Leckie, 2023
Urinary creatinine (mg/dL)	Creatinina urinară (mg/dL)	2	283.09 (127.91 - 438.27)	NC		NC	Atkins, 2021; Leckie, 2023
BUN-to-creatinine ratio	Raportul BUN-creatinină	1	15.33 (13.72 - 16.95)	NC		NC	Mansour, 2019
Serum urea (mg/dL)	Uree serică (mg/dL)	1	43.2 (38.97 - 47.43)	NC		NC	Nescolarde, 2020
Serum creatine kinase (U/L)	Creatinkinaza serică (U/L)	5	425.8 (290.88 - 560.71)	89 (77.1 - 94.7)	< 0.001	0.111	McCullough, 2011; Bekos, 2016; Mansour, 2017; Mansour, 2019; Nescolarde, 2020
TIMP-2 (ng/dL)	TIMP-2 (ng/dL)	1	12.4 (10.39 - 14.41)	NC		NC	Leckie, 2023
IGFBP-7 (ng/dL)	IGFBP-7 (ng/dL)	1	236.6 (188.33 - 284.87)	NC		NC	Leckie, 2023
TIMP-2*IGFBP (ng/dL)^2/1000	TIMP-2*IGFBP (ng/dL)^2/1000	1	4.74 (2.92 - 6.56)	NC		NC	Leckie, 2023
Urinary L-FABP (µg/g creatinine)	L-FABP urinar (ug/g creatinina)	1	2.76 (2.06 - 3.46)	NC		NC	Kosaki, 2022
Urinary NGAL (ng/mL)	NGAL urinar (ng/mL)	4	41.3 (31.74 - 50.87)	32.9 (0 - 76.2)	0.215	0.144	McCullough, 2011; Mansour, 2017; Mansour, 2019; Atkins, 2021
Plasma NGAL (ng/mL)	NGAL plasmatic (ng/mL)	2	100.68 (-5.57 - 206.94)	NC		NC	McCullough, 2011; Mansour, 2019
Serum cystatin C (mg/dL)	Cistatina serică (mg/dL)	3	0.36 (-0.11 - 0.83)	99.9 (99.9 - 99.9)	< 0.001	0.053	McCullough, 2011; Hewing, 2015; Atkins, 2021
Plasma KIM-1 (pg/mL)	KIM-1 plasmatic pg/ml	2	1682.08 (-1453.4 - 4817.57)	NC		NC	McCullough, 2011; Mansour, 2019
Urinary KIM-1 (pg/mL)	KIM-1 urinar pg/ml	3	2255.64 (827.69 - 3683.6)	92.5 (81.4 - 97)	< 0.001	0.325	McCullough, 2011; Mansour, 2017; Mansour, 2019

Serum C-reactive protein (mg/L)	Proteina C reactivă serică (mg/L)	3	0.59 (0.08 - 1.1)	95.1 (88.9 - 97.8)	< 0.001	0.908	Hewing, 2015; Bekos, 2016; Nescolarde, 2020
Copeptin (pmol/L)	Copeptina, pmol/L	1	47.53 (24.86 - 70.2)	NC		NC	Mansour, 2019
Plasma TNF-alpha (pg/mL)	TNF-alpha plasmatic (pg/mL)	1	2.7 (2.43 - 2.97)	NC		NC	Mansour, 2019
Urinary TNF-alpha (pg/mL)	TNF-alpha urinar (pg/mL)	1	0.1 (0.05 - 0.15)	NC		NC	Mansour, 2017
Plasma MCP-1 (pg/mL)	MCP-1 plasmatic pg/ml	1	392.31 (335.95 - 448.66)	NC		NC	Mansour, 2019
Urinary MCP-1 (pg/mL)	MCP-1 urinar pg/ml	2	574.3 (79.02 - 1069.59)	NC		NC	Mansour, 2017; Mansour, 2019
Plasma YKL-40 (ng/mL)	YKL-40 plasmatic ng/ml	1	40.04 (34.19 - 45.9)	NC		NC	Mansour, 2019
Urinary YKL-40 (pg/mL)	YKL-40 urinar pg/ml	2	1530.34 (361.32 - 2699.36)	NC		NC	Mansour, 2017; Mansour, 2019

CI, confidence interval; NC, not calculable. BUN, Blood Urea Nitrogen; TIMP-2, Tissue Inhibitor of Metalloproteinases-2; IGFBP-7, Insulin-like Growth Factor Binding Protein-7; L-FABP, Liver-type fatty acid binding protein; NGAL, Neutrophil Gelatinase-Associated Lipocalin; KIM-1, Kidney Injury Molecule-1; TNF-alpha, Tumor Necrosis Factor Alpha; MCP-1, Monocyte Chemoattractant Protein-1; YKL-40, Chitinase 3-like Protein 1.

Supplementary Table S4. Results of the meta-analyses of mean biomarker values 24h after the marathon, including the inconsistency index (I^2), associated p-value, Egger's test for publication bias, and the number of studies included for each biomarker.

**Biomarker**	**Biomarker**	**Number studies**	**Mean (95% CI)**	**I^2 (95% CI)**	**p**	**Egger test**	**Studies**
Serum creatinine (mg/dL)	Creatinina serică (mg/dL)	6	1.12 (0.64 - 1.61)	98.2 (97.4 - 98.8)	< 0.001	0.04	McCullough, 2011; Mansour, 2017; Mansour, 2019; Atkins, 2021; Kosaki, 2022; Leckie, 2023
Urinary creatinine (mg/dL)	Creatinina urinară (mg/dL)	2	103.74 (79.5 - 127.97)	NC		NC	Atkins, 2021; Leckie, 2023

BUN-to-creatinine ratio	Raportul BUN-creatinină	1	19.33 (16.75 - 21.92)	NC		NC	Mansour, 2019
Serum urea (mg/dL)	Uree serică (mg/dL)	0	-	-	-	-	-
Serum creatine kinase (U/L)	Creatinkinaza serică (U/L)	3	1128.14 (434.02 - 1822.26)	84.3 (53.2 - 94.8)	0.002	0.067	McCullough, 2011; Mansour, 2017; Mansour, 2019
TIMP-2 (ng/dL)	TIMP-2 (ng/dL)	1	3.2 (2.26 - 4.14)	NC		NC	Leckie, 2023
IGFBP-7 (ng/dL)	IGFBP-7 (ng/dL)	1	36.5 (21.27 - 51.73)	NC		NC	Leckie, 2023
TIMP-2*IGFBP (ng/dL)^2/1000	TIMP-2*IGFBP (ng/dL)^2/1000	1	0.18 (0.07 - 0.29)	NC		NC	Leckie, 2023
Urinary L-FABP (µg/g creatinine)	L-FABP urinar (ug/g creatinina)	1	0.63 (0.38 - 0.88)	NC		NC	Kosaki, 2022
Urinary NGAL (ng/mL)	NGAL urinar (ng/mL)	3	29.57 (-0.81 - 59.94)	99.5 (99.2 - 99.6)	< 0.001	0.451	McCullough, 2011; Mansour, 2017; Atkins, 2021
Plasma NGAL (ng/mL)	NGAL plasmatic (ng/mL)	1	10.7 (7.8 - 13.6)	NC		NC	McCullough, 2011
Serum cystatin C (mg/dL)	Cistatina serică (mg/dL)	2	0.1 (0.06 - 0.15)	NC		NC	McCullough, 2011; Atkins, 2021
Plasma KIM-1 (pg/mL)	KIM-1 plasmatic pg/ml	1	2700 (2072.81 - 3327.19)	NC		NC	McCullough, 2011
Urinary KIM-1 (pg/mL)	KIM-1 urinar pg/ml	2	1630.76 (-367.39 - 3628.9)	NC		NC	McCullough, 2011; Mansour, 2017
Serum C-reactive protein (mg/L)	Proteina C reactivă serică (mg/L)	0	-	-	-	-	-
Copeptin (pmol/L)	Copeptina, pmol/L	1	4.44 (3.05 - 5.83)	NC		NC	Mansour, 2019
Plasma TNF-alpha (pg/mL)	TNF-alpha plasmatic (pg/mL)	0	-	-	-	-	-
Urinary TNF-alpha (pg/mL)	TNF-alpha urinar (pg/mL)	1	0.02 (0.01 - 0.03)	NC		NC	Mansour, 2017
Plasma MCP-1 (pg/mL)	MCP-1 plasmatic pg/ml	0	-	-	-	-	-

Urinary MCP-1 (pg/mL)	MCP-1 urinar pg/ml	1	202.98 (100.04 - 305.91)	NC		NC	Mansour, 2017
Plasma YKL-40 (ng/mL)	YKL-40 plasmatic ng/ml	0	-	-	-	-	-
Urinary YKL-40 (pg/mL)	YKL-40 urinar pg/ml	1	219.23 (105.68 - 332.78)	NC		NC	Mansour, 2017

CI, confidence interval; NC, not calculable; BUN, Blood Urea Nitrogen; TIMP-2, Tissue Inhibitor of Metalloproteinases-2; IGFBP-7, Insulin-like Growth Factor Binding Protein-7; L-FABP, Liver-type fatty acid binding protein; NGAL, Neutrophil Gelatinase-Associated Lipocalin; KIM-1, Kidney Injury Molecule-1; TNF-alpha, Tumor Necrosis Factor Alpha; MCP-1, Monocyte Chemoattractant Protein-1; YKL-40, Chitinase 3-like Protein 1.
